# Predictors, management and prognosis of initial hyperemia of free flap

**DOI:** 10.1038/s41598-024-53834-2

**Published:** 2024-02-16

**Authors:** Juyoung Bae, Kyeong-Tae Lee

**Affiliations:** Department of Plastic Surgery, Samsung Medical Center, Sungkyunkwan University School of Medicine, 81 Irwon-ro, Gangnam-gu, Seoul, 06351 Korea

**Keywords:** Outcomes research, Risk factors, Signs and symptoms

## Abstract

In free flap operation, temporary hyperemia of the transferred flaps can often be encountered in the early postoperative period, appearing reddish and rapid capillary refilling time, which mimics venous congestion. This study aimed to investigate the factors associated with the development of hyperemia and evaluate clinical course. Consecutive patients who underwent free flap-based reconstruction between December 2019 and October 2021 were reviewed. Independent risk factors associated with its development were assessed. Flap showing initial hyperemic features were assessed using flap blood glucose measurement (BGM). If it showed over 60 mg/dL, they were closely observed without management. Their clinical outcomes were evaluated. In total, 204 cases were analyzed, of which 35 (17.2%) showed initial hyperemia. Multivariable analyses showed that using thoracodorsal artery perforator flaps and muscle containing flaps (musculocutaneous/muscle-chimeric flaps) and conducting end-to-end arterial anastomosis (vs. end-to-side) were independent predictors. All cases with initial hyperemia showed over 60 mg/dL in BGM. The phenomenon resolved spontaneously within 6.9 h averagely. Overall perfusion-related complications developed in 10 (4.9%) cases, which rate did not differ between the two groups. Several factors might be associated with the development of initial hyperemia after free flap surgery. With proper assessment, this condition can be successfully managed without unnecessary intervention.

## Introduction

Since the introduction of microsurgery in the 1950s^1^, free tissue transfer for reconstruction has constantly evolved. Owing to accumulation of anatomical knowledge and refinement of surgical techniques, the success rate of free flap transfers has dramatically improved, reaching 95 percent or more^2^. Accordingly, this has become a reliable option for the reconstruction of a diverse range of defects.

With increasing popularity, several physiological changes related to free tissue transfer and hemodynamic mechanisms have been investigated. Initial hyperemia is a transient reddish color change in the transferred flap and can develop immediately after resuming tissue perfusion via microvascular anastomosis. Several experimental and clinical studies have investigated the underlying hemodynamic mechanism for this unusual condition^3,4^. It is likely to be an adaptation process to overcome a transiently enhanced blood inflow after anastomosis, or may be attributable to temporary spams of the pedicle vessels^5^; however, much remains unknown.

This phenomenon has drawn great attention from reconstructive surgeons because its clinical features, such as the reddish discoloration of the skin and rapid capillarity refilling time (CRT), may mimic early venous insufficiency that requires emergent re-explorations and can result in serious complications including total flap failure. Physical examination is currently the gold standard for flap monitoring. However, on clinical evaluation, it may be difficult to distinguish initial hyperemia from true venous congestion, particularly for inexperienced surgeons. If venous congestion is misdiagnosed as temporary hyperemia, active intervention to salvage the flap may be delayed, possibly resulting in flap failure. Meanwhile, if the initial hyperemia is mistaken for venous insufficiency, unnecessary surgical intervention, such as flap re-exploration, may be performed, which may add to patient morbidities. A deep understanding of this phenomenon, accurate diagnosis, and proper treatments are crucial.

Despite extensive investigations, our understanding of hyperemia remains unclear. Potential predictors of the development of initial hyperemia, for example, have not been investigated because this phenomenon is not frequently visible in all flaps. In addition, appropriate methods for the differential diagnosis of true venous congestion, clinical course, and management have not been evaluated in a large clinical series. Therefore, the present study aimed to evaluate the incidence of initial hyperemia and investigate the potential predictors associated with its development. In addition, we aimed to review our experience with its management and clinical course and to evaluate the outcomes.

## Patients and methods

Based on a prospectively collected database, patients who underwent free flap-based reconstruction between December 2019 and October 2021 by a single surgeon were identified. Patients with missing data for the pertinent variables were excluded. This study was conducted with the approval of the institutional review board of Samsung Medical Center (IRB No. 2023-03-028) and adhered to the tenets of the Declaration of Helsinki. The requirement for written informed consent was waived due to the retrospective nature of the study.

### Detection and management of initial hyperemia

Flap monitoring was performed according to a predetermined standard protocol at our department and conducted based on clinical evaluations with the aid of a portable Doppler. (ES-100V3, Hadeco, Japan) Photographs of the flap’s current appearance, surface temperature, Doppler sound of the perforators, and CRT were regularly checked by ancillary doctors and shared through a private mobile messenger application among the attending surgeons and residents involved in the surgery. Monitoring was initiated immediately after the patient returned to the ward, and regularly conducted by on-duty junior residents every 3 h for the first 24 h after the operation. Initial hyperemia was defined as flaps showing reddish discoloration and rapid CRT for less than 1.5 s immediately after resuming tissue perfusion via microvascular anastomosis (see supplementary Video S1 online, this video was recorded during flap monitoring immediately following surgery. The flap is red in color and demonstrates reduced CRT).

For flaps showing initial hyperemic features, blood glucose measurements (BGM) were conducted on the flap to distinguish between initial hyperemia and venous congestion during the first flap monitoring. The reliability of evaluating the perfusion status of flaps by measuring BGM has been the subject of several experimental and clinical studies recently^6–8^. Although glucose levels can be influenced by various factors, such as stress, it has been known that a significant decrease in glucose levels in a flap could indicate excessive glucose consumption related to inadequate venous outflow, suggesting the development of venous congestion. As reported in the literature, considering a judgment threshold of 60 mg/dL^6^, if the level of flap BGM was more than the level, we determined that the flap did not exhibit a true venous insufficiency state; instead, we considered it to be in a state of initial hyperemia with smooth venous outflow and conducted routine observation in the same manner as flaps with normal clinical features. Conversely, when the flap BGM was below 60, we characterized the condition of flap as venous congestion due to impaired venous outflow, not initial hyperemia, and implemented appropriate interventions, including re-exploration.

### Data collection and outcome measure

Data were retrieved from a pre-existing database, that was prospectively updated by ancillary doctors or extracted from the Clinical Data Warehouse of Samsung Medical Center for this study. The baseline demographic variables included age, body mass index (BMI), American Society of Anesthesiologists (ASA) score, history of tobacco use, comorbidities such as hypertension and diabetes mellitus, and preoperative albumin and hemoglobin levels. Collected reconstructive surgery-related data included operation time, ischemic time, etiology of defect, recipient region, flap type [perforator flap, musculocutaneous (MC) flap, muscle-chimeric flap, or osteocutaneous flap], flap kinds, donor region of the flaps, pedicle length, arterial anastomosis type, number of vein anastomosis, and size of skin flap and muscle flap. The hemodynamic parameters recorded were blood transfusion status, type of transfused blood, intraoperative fluid administration, intraoperative urine output, intraoperative net fluid balances, preoperative blood pressure, intraoperative mean noninvasive and arterial blood pressure, difference between preoperative and intraoperative mean blood pressure, and mean temperature during the operation. The net fluid balance (mg/h/kg) was calculated as intraoperative plasma volume resuscitation minus intraoperative urine output.

The primary outcome was the development of initial hyperemia as defined above. The secondary outcomes were the clinical course of the flaps showing initial hyperemia and management outcomes. The development of perfusion-related complications, including total or partial flap loss, arterial/venous insufficiency, and emergency return to the operating room due to threatened flaps, was also noted and evaluated in both groups.

### Statistical analysis

Patients with flaps showing initial hyperemia and those without flaps were compared in terms of patient- and operation-related variables. Pearson’s chi-square test or Fisher’s exact test was used to analyze categorical variables, and the Student’s t-test or Mann–Whitney test for continuous variables. To identify the independent predictors associated with the occurrence of hyperemia, univariable and multivariable logistic regression analyses were conducted by calculating the odds ratios (OR) and 95% confidence intervals (CIs). Backward selection models were chosen for the multivariable regression models. The goodness of fit of the models was examined using the Hosmer–Lemeshow test. A *p* value of less than 0.05 was considered statistically significant. All statistical analyses were conducted using IBM SPSS software (version 23.0, IBM Corp., Armonk, N.Y., USA).

## Results

During the study period, 208 flaps on 184 patients were performed. Among them, four flaps with missing data were excluded and 204 cases were finally included in the analysis. The patients’ mean age was 59.4 (13–86) years, and the mean BMI was 24.9 (13.2–35.9) kg/m^2^. Of these, 35 flaps showed initial hyperemia, with an incidence of 17.2 percent, and the other 169 did not. The hyperemia and non-hyperemia groups generally showed similar demographic characteristics regarding age, BMI, ASA classification, smoking history, and co-morbidities, except for hypertension (*p* value = 0.046) (Table 1).

**Table 1:** Patients’ demographics of the two groups.

Variables	Overall (N = 204, 100%)	Hyperemia (N = 35, 17.2%)	No hyperemia (N = 169, 81.8%)	*p* Value
Age (years)				
Mean (SD)	59.4 (± 16.4)	59.2 (± 17.0)	59.4 (± 16.3)	0.929
Range	13–86	20–86	13–85	
BMI (kg/m^2^)	24.9 (± 4.4)	24.5 (± 3.6)	25.0 (± 4.5)	0.515
ASA class				0.692
I	25 (12.3%)	3 (8.6%)	22 (13.0%)	
II	143 (70.1%)	26 (74.3%)	117 (69.2%)	
III	32 (15.7%)	6 (17.1%)	26 (15.4%)	
IV	4 (2.0%)	0 (0.0%)	4 (2.4%)	
Diabetes	55 (27.0%)	6 (17.1%)	49 (29.0%)	0.150
HTN	77 (37.7%)	8 (22.9%)	69 (40.8%)	0.046
Smoking history				0.130
Never/Former	192 (94.1%)	31 (88.6%)	161 (95.3%)	
Active	12 (5.9%)	4 (11.4%)	8 (4.7%)	
Preoperative hemoglobin	12.8 (11.1, 14.2)	13.4 (12.0, 14.3)	12.7 (10.7, 14.2)	0.314
Preoperative albumin	4.3 (3.9, 4.6)	4.2 (3.8, 4.7)	4.3 (3.9, 4.6)	0.937

*ASA class* American society of anesthesiologists physical status classification.

**Figure 1:**
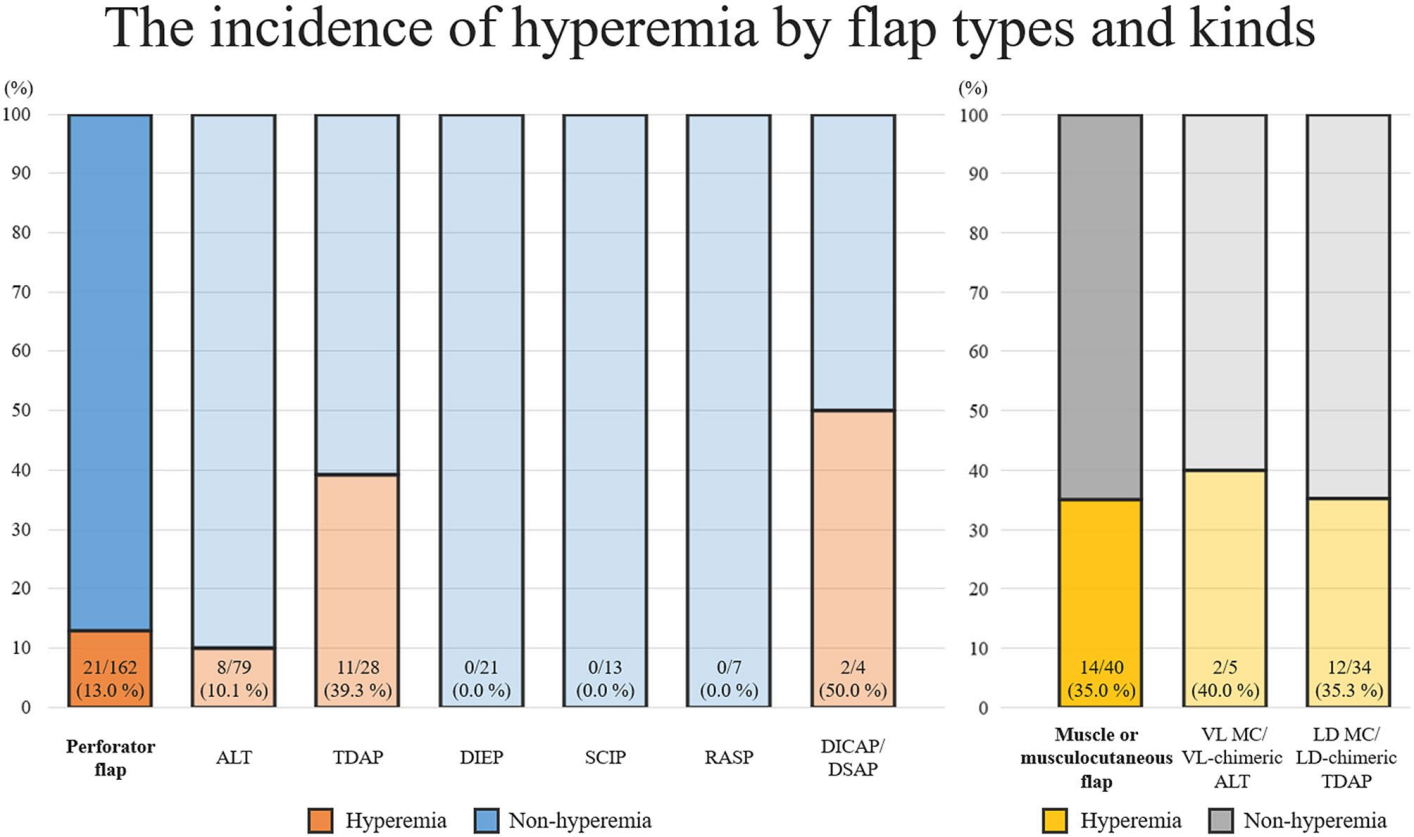
The rates of initial hyperemia according to the flap types. ALT: Anterolateral thigh, TDAP: Thoracodorsal artery perforator flap, DIEP: Deep inferior epigastric perforator, SCIP: Superficial circumflex iliac artery perforator, RASP: Radial artery superficial palmar branch, DSAP: Dorsal scapular artery perforator, DICAP: Dorsal intercostal artery perforator, VL: Vastus lateralis, MC: musculocutaneous, LD: Latissimus dorsi.

The reconstructive operation-related data are summarized in Table 2. In the overall study population, the majority of defects were oncologic resections (67.5%), and the trunk and head and neck regions were the most common recipient sites. The perforator flap was the most commonly used flap type, of which the anterolateral thigh (ALT) perforator flaps were the most predominant. In the comparison between the initial hyperemia and the non-hyperemia groups, the types and specific kinds of flaps, donor regions, and size of the skin paddle of the flaps (length, width, and dimension) differed significantly. The musculocutaneous (MC) and muscle-chimeric flaps showed higher rates of initial hyperemia than the perforator flap. Regarding the specific kinds of flaps, Vastus lateralis (VL) MC/VL-chimeric ALT, thoracodorsal artery perforator (TDAP), latissimus dorsi (LD) MC/LD-chimeric TDAP, and dorsal intercostal artery perforator (DICAP)/dorsal scapular artery perforator (DSAP) flaps showed higher rates of initial hyperemia than the other kinds of flaps (Fig. 1). The mean size of flaps regarding their length and width was significantly larger in the hyperemia group than in the non-hyperemia group. Cases using end-to-end arterial anastomosis tended to show a higher rate of initial hyperemia than those using end-to-side, which difference was marginally significant (*p* value = 0.055).

**Table 2:** Reconstructive operation related variables of the two groups.

Variables	Overall (N = 204, 100%)	Hyperemia (N = 35, 17.2%)	No hyperemia (N = 169, 81.8%)	*p* Value
Operation time (hour)	6.7 (5.0, 8.8)	7.0 (5.1, 8.4)	6.5 (5.0, 8.9)	0.696
Ischemic time (min)	78.5 (65.0, 96.0)	82.0 (68.0, 108.0)	78.0 (62.0, 95.0)	0.181
Etiology of defects				0.724
Cancer	138 (100.0%)	22 (15.9%)	116 (84.1%)	
Chronic wound	52 (100.0%)	9 (17.3%)	43 (82.7%)	
Trauma, scar	10 (100.0%)	3 (30.0%)	7 (70.0%)	
Congenital	3 (100.0%)	1 (33.3%)	2 (66.7%)	
Others	1 (100.0%)	0 (0.0%)	1 (100.0%)	
Recipient region				0.139
Head and neck	37 (18.3%)	8 (22.9%)	29 (17.2%)	
Trunk	41 (20.3%)	5 (14.3%)	36 (21.3%)	
Back	5 (2.5%)	0 (0.0%)	5 (3.0%)	
Upper extremity	27 (13.2%)	2 (5.7%)	24 (14.8%)	
Perineum	13 (6.4%)	5 (14.3%)	8 (4.7%)	
Lower extremity	81 (39.7%)	15 (42.9%)	66 (39.1%)	
Flap types				0.009
Perforator flap	162 (100%)	21 (13%)	141 (87%)	
Musculocutaneous flap	30 (100%)	10 (33.3%)	20 (66.7%)	
Muscle-chimeric flap	10 (100%)	4 (40%)	6 (60%)	
Osteocutaneous flap	2 (100%)	0 (0%)	2 (100%)	
Flap kinds				< 0.001
ALT	79 (100%)	8 (10.1%)	71 (89.9%)	
VL MC/VL-chimeric ALT	5 (100%)	2 (40%)	3 (60%)	
TDAP	28 (100%)	11 (39.3%)	17 (60.7%)	
LD MC/LD-chimeric TDAP	34 (100%)	12 (35.3%)	22 (64.7%)	
DIEP	21 (100%)	0 (0%)	21 (100%)	
SCIP	13 (100%)	0 (0%)	13 (100%)	
RASP	7 (100%)	0 (0%)	7 (100%)	
RFF	7 (100%)	0 (0%)	7 (100%)	
Fibular	2 (100%)	0 (0%)	2 (100%)	
DICAP/DSAP	4 (100%)	2 (50%)	2 (50%)	
Others	4 (100%)	0 (0%)	4 (100%)	
Donor region				< 0.001
Thigh	84 (100.0%)	10 (11.9%)	74 (88.1%)	
Back	67 (100.0%)	25 (37.3%)	42 (62.7%)	
Abdomen	21 (100.0%)	0 (0.0%)	21 (100.0%)	
Radial artery system	14 (100.0%)	0 (0.0%)	14 (100.0%)	
Groin	13 (100.0%)	0 (0.0%)	13 (100.0%)	
Others	5 (100.0%)	0 (0.0%)	5 (100.0%)	
Pedicle length (cm)	7.0 (5.0, 10.0)	7.0 (5.4, 11.0)	7.0 (5.0, 10.0)	0.431
Anastomosis type				0.055
End-to-end	178 (100.0%)	34 (19.1%)	144 (80.9%)	
End-to-side	26 (100.0%)	1 (3.8%)	25 (96.2%)	
Turbocharging	12 (5.9%)	1 (2.9%)	11 (6.5%)	0.695
Vein anastomosis				0.096
1 Vein	130 (100.0%)	18 (13.8%)	112 (86.2%)	
2 Veins	74 (100.0%)	17 (23.0%)	57 (77.0%)	
Skin flap size				
Length (cm)	15.0 (11.0, 20.0)	17.0 (14.5, 22.0)	14.0 (11.0, 20.0)	0.021
Width (cm)	8.0 (6.0, 10.0)	8.0 (7.5, 10.0)	7.5 (6.0, 10.0)	0.010
Dimension (length × width, cm^2^)	120.0 (71.5, 187.8)	153.0 (116.0, 220.0)	112.0 (60.3, 182.0)	0.008
Ratio (length/width)	1.9 (1.7, 2.3)	1.9 (1.7, 2.2)	2.00 (1.7, 2.4)	0.499
Muscle flap size (N = 39)				
Length (cm)	17.0 (9.0, 20.0)	17.5 (12.0, 21.0)	17.0 (8.0, 19.5)	0.481
Width (cm)	7.0 (5.0, 10.0)	7.5 (5.9, 10.8)	7.0 (5.0, 10.5)	0.724
Area (cm^2^)	128.0 (48.0, 204.0)	133.0 (66.3, 213.8)	108.0 (40.0, 219.0)	0.639

*ALT* anterolateral thigh, *VL* vastus lateralis, *MC* musculocutaneous, *TDAP* thoracodorsal artery perforator, *LD* latissimus dorsi, *DIEP* deep inferior epigastric artery perforator, *SCIP* superficial circumflex iliac artery perforator, *RASP* radial artery superficial palmar branch perforator, *RFF* radial forearm flap, *DICAP* dorsal intercostal artery perforator, *DSAP* dorsal scapular artery perforator.

There were no significant differences in the rates of transfusion, intraoperative plasma volume resuscitation, urine output, or net fluid balance between the two groups (see supplementary Table S1 online). In addition, baseline, intraoperative mean noninvasive, arterial, preoperative and intraoperative mean blood pressures, and mean temperature did not differ between the groups, except for the mean diastolic arterial blood pressure (*p* value = 0.020) (see supplementary Table S2 online).

### Univariable and multivariable analysis for predictors of initial hyperemia

In the analysis for flap types, cases using the ALT perforator flap were the most predominant, thus served as the reference. On univariable analysis, MC and muscle-chimeric flaps as a type flap, TDAP and LD musculocutaneous/LD-chimeric TDAP flap as a flap kind, and back as a donor region were associated with an increased likelihood of hyperemia. Multivariable analysis revealed that compared to the ALT perforator flap, the VL MC/VL-chimeric ALT, TDAP, and LD MC/LD-chimeric TDAP flap were significantly associated with an increased rate of initial hyperemia after adjusting for other variables (Table 3). End-to-end anastomoses was also an independent risk factor for the development of hyperemia compared to conducting end-to-side anastomosis. Other variables, including ischemic time, flap size, number of venous anastomoses, and all hemodynamic variables, did not significantly influence the outcome.

**Table 3:** Univariable and multivariable analysis to identify predictors for initial hyperemia.

Variables	UVA OR (95% CI)	Unadjusted *p* Value	MVA OR (95% CI)	Adjusted *p* Value
Age	1.00 (0.98, 1.02)	0.929		
BMI	0.97 (0.89, 1.06)	0.513		
Diabetes mellitus	0.51 (0.20, 1.30)	0.156		
Hypertension	0.43 (0.18, 1.00)	0.050	0.46 (0.11, 0.46)	0.109
Smoking history				
Never/Former	Ref			
Active	2.60 (0.74, 9.16)	0.138		
Ischemic time (min)	1.01 (1.00, 1.02)	0.239		
Etiology of defects				
Cancer	Ref	0.780		
Chronic wound	1.10 (0.47, 2.58)	0.820		
Trauma, scar	2.26 (0.54, 9.42)	0.263		
Congenital	2.64 (0.23, 30.35)	0.437		
Others	0.00 (0.00, .)	1.000		
Recipient region				
Head and neck	Ref	0.370		
Trunk	0.50 (0.15, 1.71)	0.270		
Back	0.00 (0.00, .)	0.999		
Upper extremity	0.29 (0.06, 1.49)	0.139		
Perineum	2.27 (0.58, 8.87)	0.240		
Lower extremity	0.82 (0.32, 2.16)	0.693		
Flap name				
ALT	Ref		Ref	
VL MC/VL-chimeric ALT	5.92 (0.86, 40.88)	0.071	11.43 (0.04, 11.43)	0.037
TDAP	5.74 (2.00, 16.47)	0.001	6.00 (0.00, 6.00)	0.002
LD MC/LD-chimeric TDAP	4.84 (1.76, 13.35)	0.002	4.36 (0.01, 4.36)	0.006
DIEP	0.00 (0.00, .)	0.998	0.00 (1.00, 0.00)	0.998
SCIP	0.00 (0.00, .)	0.999	0.00 (1.00, 0.00)	0.999
RASP	0.00 (0.00, .)	0.999	0.00 (1.00, 0.00)	0.999
RFFF	0.00 (0.00, .)	0.999	0.00 (1.00, 0.00)	0.999
Fibular	0.00 (0.00, .)	0.999	0.00 (1.00, 0.00)	0.999
DICAP/DSAP	8.88 (1.10, 71.89)	0.041	8.22 (0.05, 8.22)	0.054
Others	0.00 (0.00, .)	0.999	0.00 (1.00, 0.00)	0.999
Donor region				
Thigh	Ref	0.030		
Back	4.41 (1.93, 10.06)	< 0.001		
Abdomen	0.00 (0.00, .)	0.998		
Radial artery system	0.00 (0.00, .)	0.999		
Groin	0.00 (0.00, .)	0.999		
Others	0.00 (0.00, .)	0.999		
Turbocharging	0.42 (0.05, 3.38)	0.417		
Anastomosis type				
End-to-end	Ref		Ref	
End-to-side	0.17 (0.02, 1.29)	0.087	0.10 (0.03, 0.10)	0.033
Number of vein anastomosis				
1 Vein	Ref			
2 Veins	1.86 (0.89, 3.87)	0.099		
Skin flap size				
Length (cm)	1.11 (0.99, 1.24)	0.067		
Width (cm)	1.02 (0.98, 1.07)	0.266		
Dimension (length × width, cm^2^)	1.00 (1.00, 1.00)	0.402		
Ratio (length/width)	0.90 (0.47, 1.73)	0.748		
Red blood cell transfusion	1.08 (0.34, 3.42)	0.893		
Plasma volume resuscitation (ml/h/kg)	0.81 (0.53, 1.25)	0.349		
Urine output (ml/h/kg)	0.86 (0.62, 1.21)	0.384		
ABP systolic	0.98 (0.95, 1.01)	0.140		
ABP mean	1.00 (0.96, 1.05)	0.951		
ABP diastolic	1.03 (0.97, 1.08)	0.338		

*ALT* anterolateral thigh, *VL* vastus lateralis, *MC* myocutaneous, *TDAP* thoracodorsal artery perforator, *LD* latissimus dorsi, *DIEP* deep inferior epigastric artery perforator, *SCIP* superficial circumflex iliac artery perforator, *RASP* radial artery superficial palmar branch perforator, *RFF* radial forearm flap, *DICAP* dorsal intercostal artery perforator, *DSAP* dorsal scapular artery perforator, *ABP* arterial blood pressure.

### Subgroup analysis for the perforator flap

As the perforator flap was the most predominant type of flap used, additional analyses were conducted to identify the independent predictors of initial hyperemia in this subgroup. Similar results also showed that the TDAP and DICAP/DSAP flaps were significantly associated with the development of initial hyperemia compared to that of the ALT perforator flap in the multivariable analyses. The method of arterial anastomosis also significantly influenced the outcome. Additionally, the dimensions of the skin flaps were significantly associated with the development of initial hyperemia, showing that harvesting a larger dimension of the skin paddle increased the risk for hyperemia after adjusting for other variables (Table 4).

**Table 4:** A subgroup analysis of the occurrence of hyperemia following perforator flap surgery.

Variables	UVA OR (95% CI)	*p* Value	MVA OR (95% CI)	*p* Value
Age	1.00 (0.97, 1.03)	0.869		
BMI	1.01 (0.90, 1.12)	0.931		
Diabetes mellitus	0.62 (0.20, 1.94)	0.408		
Hypertension	0.47 (0.16, 1.37)	0.168		
Smoking history				
Never/former	Ref			
Active	0.00 (0.00, .)	0.999		
Ischemic time (min)	1.01 (0.99, 1.02)	0.426		
Wound cause				
Cancer	Ref	0.587		
Chronic wound	0.74 (0.20, 2.75)	0.656		
Trauma, scar	1.98 (0.38, 10.44)	0.420		
Congenital	3.47 (0.30, 40.61)	0.322		
Recipient region				
Head and neck	Ref		Ref	
Trunk	0.51 (0.14, 1.95)	0.329	2.66 (0.42, 16.89)	0.301
Back	0.00 (0.00, .)	0.999	0.00 (0.00, .)	0.999
Upper extremity	0.32 (0.06, 1.70)	0.182	2.42 (0.24, 24.72)	0.455
Perineum	0.00 (0.00, .)	0.999	0.00 (0.00, .)	0.999
Lower extremity	0.57 (0.19, 1.74)	0.325	0.52 (0.13, 2.00)	0.338
Flap name				
ALT	Ref		Ref	
TDAP	5.74 (2.00, 16.47)	0.001	5.90 (1.48, 23.49)	0.012
DIEP	0.00 (0.00, .)	0.998	0.00 (0.00, .)	0.998
SCIP	0.00 (0.00, .)	0.999	0.00 (0.00, .)	0.999
RASP	0.00 (0.00, .)	0.999	0.00 (0.00, .)	0.999
RFFF	0.00 (0.00, .)	0.999	0.00 (0.00, .)	0.999
DICAP/DSAP	8.88 (1.10, 71.89)	0.041	17.51 (1.23, 248.99)	0.035
Others	0.00 (0.00, .)	0.999	0.00 (0.00, .)	0.999
Donor region				
Thigh	Ref	0.042		
Back	5.77 (2.10, 15.85)	0.001		
Abdomen	0.00 (0.00, .)	0.998		
Radial artery system	0.00 (0.00, .)	0.999		
Groin	0.00 (0.00, .)	0.999		
Others	0.00 (0.00, .)	0.999		
Turbocharging	0.00 (0.00, .)	0.999		
Anastomosis type				
End-to-end	Ref			
End-to-side	0.30 (0.04, 2.38)	0.256	0.14 (0.01, 1.63)	0.117
Number of vein anastomosis				
1 Vein	Ref			
2 Veins	2.00 (0.79, 5.07)	0.142		
Skin flap size				
Length (cm)	1.16 (1.00, 1.33)	0.045		
Width (cm)	1.01 (0.96, 1.06)	0.675		
Dimension (length × width, cm^2^)	1.00 (1.00, 1.00)	0.612	1.01 (1.00, 1.02)	0.024
Ratio (length / width)	0.45 (0.16, 1.22)	0.115		
Transfusion (RBC)	1.24 (0.26, 6.05)	0.787		
Plasma volume resuscitation (ml/h/kg)	0.61 (0.27, 1.38)	0.236		
Urine output (ml/h/kg)	0.72 (0.43, 1.22)	0.217		
ABP systolic	0.97 (0.94, 1.01)	0.125		
ABP mean	0.98 (0.94, 1.03)	0.491		
ABP diastolic	1.00 (0.94, 1.06)	0.985		

Univariable and multivariable analysis for identifying predictors of initial hyperemia in cases using perforator flaps.

*ALT* anterolateral thigh, *TDAP* thoracodorsal artery perforator, *DIEP* deep inferior epigastric artery perforator, *SCIP* superficial circumflex iliac artery perforator, *RASP* radial artery superficial palmar branch perforator, *RFF* radial forearm flap, *DICAP* dorsal intercostal artery perforator, *DSAP* dorsal scapular artery perforator, *ABP* arterial blood pressure.

### Clinical course of initial hyperemia

**Figure 2:**
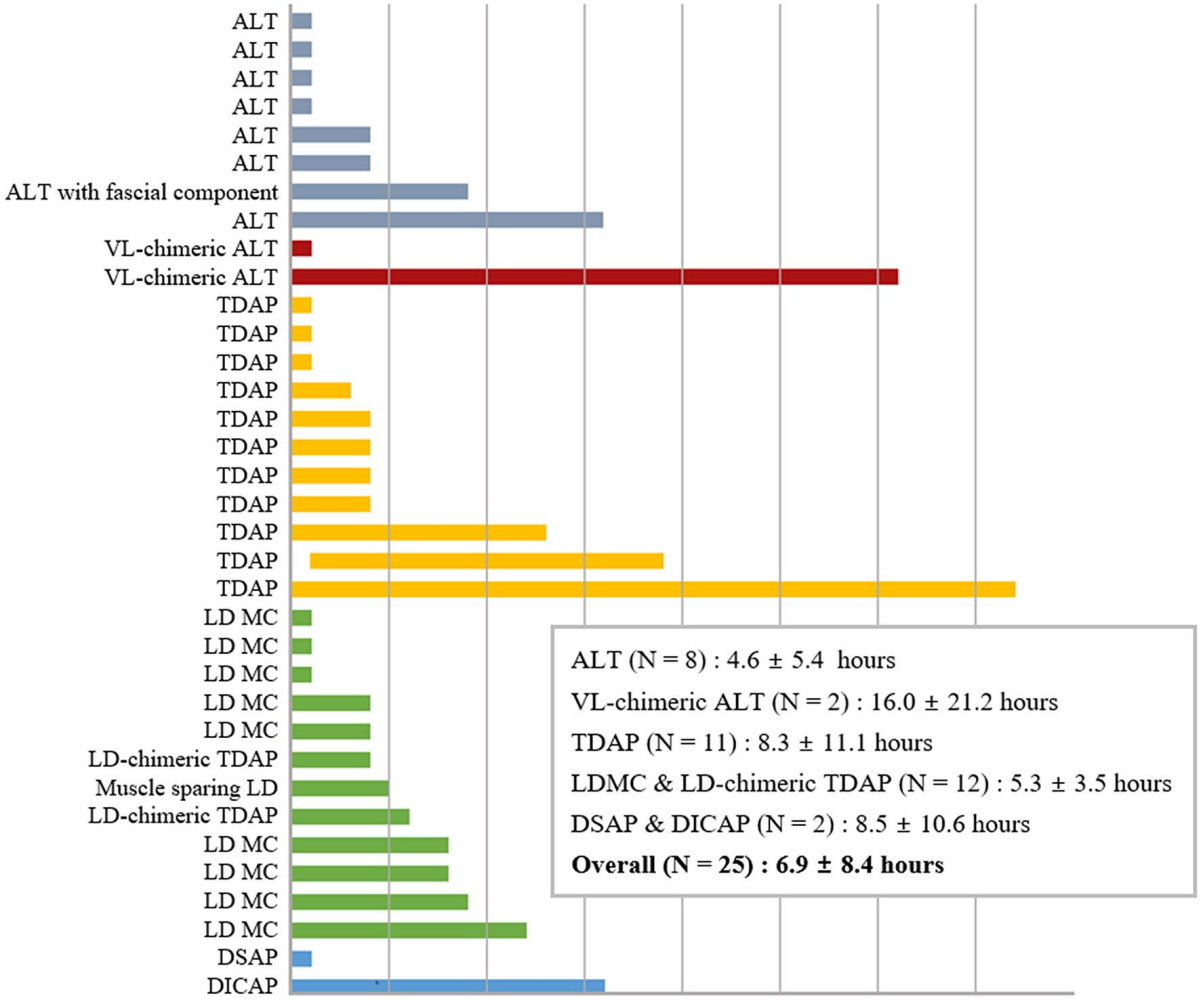
The onset and resolution time of initial hyperemia for each flap. The average resolution times by flap type are presented in the box. ALT: Anterolateral thigh, VL: Vastus lateralis, TDAP: Thoracodorsal artery perforator, LD: Latissimus dorsi, MC: musculocutaneous, DSAP: Dorsal scapular artery perforator, DICAP: Dorsal intercostal artery perforator.

All flaps in the initial hyperemia group showed a BGM level of > 60 mg/dL during initial flap monitoring, with a mean of 106 mg/dL (range, 89–134 mg/dL). Initial hyperemia resolved spontaneously after a few hours. Figure 2 shows the onset and resolution times of initial hyperemia. The average resolution time was 6.9 ± 8.4 h. The average time for hyperemia resolution was the longest in DSAP and DICAP flaps, of 8.5 h, and the shortest time observed in the LD MC flap, of 5.3 h. However, the resolution time did not differ significantly between the flap kinds. A representative case is shown in supplementary Video S2. (see supplementary Video S2 online. This video demonstrates flaps with hyperemic features immediately following surgery. After eight hours, the hyperemic characteristics of the flaps disappeared.)

### Perfusion-related complications

Overall, perfusion-related complications developed in 10 cases (4.9%), four and six of which were related to arterial insufficiency and venous insufficiency, respectively. Total and partial flap loss occurred in four (2.0%) and seven (3.4%) cases, respectively. All but one of the cases that developed venous insufficiency had no clinical features of initial hyperemia in the immediate postoperative period. The rates of overall perfusion-related complications and each type independently were not significantly different between the hyperemia and the non-hyperemia groups (Table 5).

**Table 5:** Comparison of perfusion-related complications between the two groups.

	Overall (N = 204, 100%)	Hyperemia (N = 35, 17.2%)	No hyperemia (N = 169, 81.8%)	*p* Value
Perfusion-related complication	10 (4.9%)	1 (2.9%)	9 (5.3%)	1.000
Arterial insufficiency	4 (2.0%)	0 (0.0%)	4 (2.4%)	1.000
Venous insufficiency	6 (2.9%)	1 (2.9%)	5 (3.0%)	1.000
Re-operation	43 (21.1%)	5 (13.2%)	38 (22.9%)	0.269
Flap revision	6 (2.9%)	1 (2.9%)	5 (3.0%)	1.000
Hematoma evacuation	5 (2.5%)	0 (0.0%)	5 (3.0%)	0.590
Wound revision	35 (17.2%)	5 (14.3%)	30 (17.8%)	0.621

## Discussion

Initial hyperemia following free flap surgery is a concerning condition that causes confusion to operators and may prompt emergency reoperations because of its clinical features similar to venous congestion. The present study evaluated the incidence, detection, and treatment outcomes of initial hyperemia in 204 consecutive patients who underwent free flap reconstruction for a diverse range of defects. Risk factors for the development of hyperemia were identified, and the association between hyperemia and complications was investigated. To the best of our knowledge, the present study is the largest series to focus on the clinical outcomes of hyperemia in various flaps and defects. A flap failure rate of 2% was observed in this series of free flap reconstructions, including head and neck and lower extremity reconstruction. This result is similar to those of previous studies^2,9^, indicating that free flap reconstruction is a safe and reliable method.

In the current study, the incidence of initial hyperemia was 17.2 percent, which was higher than expected. The rate of hyperemia in our study is similar to those of previous reports on LD MC and TDAP flaps (19.8%)^5^, and DIEP flaps (13.7%)^10^. It can be assumed that the development of initial hyperemia may not be uncommon after free tissue transfer. This may imply the clinical importance of this phenomenon in free flap-based reconstruction, and reconstructive surgeons should be aware that initial hyperemia often develops in the early postoperative period. Moreover, accurate diagnosis and proper management of hyperemia may be crucial for achieving optimal outcomes.

We found that the incidence of hyperemia was significantly higher in the TDAP, LD -chimeric TDAP, LD MC, and DICAP/DSAP flaps than in the other flaps. Interestingly, these flaps shared the same donor site in the back region. In another study reporting temporary vascular insufficiency, initial hyperemia was also reported more frequently in LD and TDAP flaps than in other flaps^5^, consistent with our findings. Although the exact mechanism for this finding could not be identified in this clinical study, some clue could be found in previous anatomical studies reporting that the subcutaneous course and spreading patterns of perforators may differ according to the type of perforator flap^11,12^. There are three types of perforator morphologic patterns. The type 1 perforator penetrates almost perpendicularly into the subdermal plexus. A type 2 perforator protrudes from the residual fat with a diameter of two centimeters, branching off into the adipose tissue. A type 3 perforator extends across the deep fascia and gradually penetrates the adipose tissue^11^. In a computed tomographic angiogram study, a type 1 perforator morphological pattern was found in 33 percent of ALT flaps^13^, while in 50 percent of TDAP flaps^12^. This suggests that tissue perfusion of these flaps may occur predominantly at the level of the subdermal plexus or superficial adipose layer, subsequently leading to showing the high incidence of hyperemia. Further experimental and dynamic anatomical studies are required.

There was also a high incidence of hyperemia in the MC and muscle-chimeric flap groups in our study. This suggests that flaps containing muscle components could have higher risks of developing initial hyperemia than perforator flaps containing only skin and subcutaneous tissue. Reportedly, vascular resistance and microcirculation differ according to the flap entities^14^, and the type^15–17^. Consequently, blood inflow to the flap could differ according to the flap type. That is, the amount of blood flow to MC flaps after anastomosis could be greater than that to other types of flaps^16,18,19^. Also, in the present study, when the analysis was restricted to cases using perforator flaps, an increase in skin flap size was associated with an increase in the incidence of hyperemia. This may be attributed to the fact that, as the tissue component volume increases, vascular resistance decreases and blood flow increases^20,21^, which may increase the risk of hyperemia.

In the present study, cases with end-to-end anastomosis tended to have higher odds of initial hyperemia compared to those with end-to-side. There is currently a lack of quantitative studies objectively measuring hemodynamics including blood flow between end-to-end and end-to-side anastomoses. Nevertheless, it may be feasible to infer indirectly through various experimental and clinical studies. An animal study indicates that the angle of anastomosis in end-to-side procedures influences arterial flow, showing that artery connected with an angle close to 90 degrees resulted in relatively lower flow, while angles closer to parallel showed higher flow^22^. In recent clinical studies, quantitative measurements were conducted on the blood flow directed towards the flap and the flow towards the distal area after performing end-to-side anastomosis^18,23^. Although variations were observed based on the anastomotic angle, the blood flow entering the flap was consistently lower than the flow directed distally. This suggests that, in the context of end-to-side anastomosis, the blood flow towards the flap may be comparatively reduced compared to that in end-to-end anastomosis. Although further studies would be required, it is plausible to consider that end-to-end anastomosis may lead to increased initial artery inflow, potentially elevating the risk of hyperemia compared to end-to-side.

We observed that the arteriovenous anastomosis ratio, specifically the number of venous anastomoses, showed a significant association with the occurrence of initial hyperemia in the univariable analysis. It is likely that the increased number of anastomosed veins may facilitate venous outflow and enhance the effective resolution of early hyper-inflow after the anastomosis. Additionally, considering the structure of the perforator complex, which is composed of one artery and two veins, this ratio might align more closely with physiological conditions. Further investigation may be warranted to explore this presumption.

Diverse hemodynamic variables were not associated with the development of hyperemia. Indeed, a simple comparison of mean diastolic arterial blood pressure (ABP) between the group experiencing initial hyperemia and the group without showed a difference. However, when examining the association between diastolic ABP and the occurrence of initial hyperemia through logistic regression analysis, both univariable and multivariable analyses did not yield statistical significance. From this, it can be inferred that the difference in diastolic ABP between the two groups may be attributed to other confounding factors. In contrast, hypertension was significantly more prevalent in the non-hyperemia group. A key pathological hallmark of hypertension is increased peripheral vascular resistance^24^, which may have a protective effect on the transient blood flow increase after anastomosis in patients with hypertension.

In this study, flap BGM was performed when the flap was suspected to have hyperemia to distinguish hyperemia from venous congestion. Blood glucose levels in the flaps are sensitive and specific indicators of flap venous congestion^25,26^. However, flap BGM has not been widely used to detect hyperemia. In this study, hyperemia was diagnosed when the flap BGM levels were > 60 mg/dL. Based on this criterion, no false-positive or false-negative results were observed. Therefore, we can assume that flap BGM could be a reliable tool for detecting hyperemia and venous congestion.

Several experimental studies have indicated that blood flow to the flap increases after anastomosis and subsequently decreases after day one^14,27^. This transient increase in blood flow could result in hyperemia during the first 24 h after anastomosis. In this study, most cases of hyperemia resolved spontaneously within one day after anastomosis, indicating that increased blood flow immediately after anastomosis may be responsible for the occurrence of hyperemia. It has been suggested that T-anastomosis, additional venous anastomosis, and supercharging may protect against the development of hyperemia^5^. However, hyperemia is a physiological phenomenon that occurs after anastomosis and resolves without causing complications, as shown here. Therefore, no additional procedures were required to prevent hyperemia. Hyperemia can be diagnosed using BGM, and if the level is greater than 60 mg/dL, the patient should be reassured while the flap condition is monitored closely.

This study has some limitations, many of which are inherent to its retrospective design. Data on muscle size were unavailable for some flaps and the weight of the flap could not be obtained. Moreover, the assessment of hyperemia in this study may have been subjective to some degree. However, to objectively assess and record hyperemia as much as possible, the flap status was shared in real time with at least six doctors for each flap and evaluated through discussion, which may minimize this confounding issue. The heterogeneity in flap type, variation in defect etiology and recipient site stands as one of the significant limitations of our study. Although we attempted to adjust for these factors through multivariable analysis, further research under more controlled conditions may be necessary. Lastly, although we investigated the risk factors and prognosis of hyperemia, the pathophysiology of hyperemia is still unclear. Further anatomical and experimental research is required to understand the mechanisms underlying hyperemia in detail.

## Conclusion

The results of this retrospective cohort study suggest that initial hyperemia may develop frequently after free flap-based reconstruction. Several patients- and operation-related characteristics, including hypertension, type and skin paddle size of the harvested flaps, and the manner of arterial anastomosis, may be associated with the development of initial hyperemia. In addition, hyperemia may not be a pathologic condition or prodromal symptom of venous insufficiency, and may not result in flap-threatening. Instead, it could be considered a physiologic phenomenon. That is, hyperemia can resolve spontaneously mostly within one day postoperatively, and may not require any intervention such as surgical re-exploration. An accurate diagnosis to differentiate true venous insufficiency is crucial, for which BGM of the flap may be helpful. This information may be useful in preoperative planning, intraoperative decision-making and postoperative management of free flap-based reconstruction.

### Supplementary Information

Supplementary Information 1.

Supplementary Information 2.

Supplementary Video 1.

Supplementary Video 2.

## Data Availability

The datasets used and/or analyzed during the current study available from the corresponding author on reasonable request.
