# Supplementary material for: Predictors, management and prognosis of initial hyperemia of free flap
Source: Sci Rep. 2024 Feb 16;14:3894. doi: 10.1038/s41598-024-53834-2 (PMC10873382; doi:10.1038/s41598-024-53834-2)
Supplement: Supplementary file 1 — Supplementary Information 1. [file 41598_2024_53834_MOESM1_ESM.docx]

**Supplementary video 1.** This video shows flaps immediately following surgery. The flaps are red in color and demonstrated reduced capillary refilling time. LD: Latissimus dorsi, TDAP: thoracodorsal artery perforator, MC: musculocutaneous

**Supplementary video 2**. A thoracodorsal artery perforator (TDAP) flap is demonstrated following the excision of the acral lentiginous melanoma. The video on the left shows a hyperemic TDAP flap immediately after surgery, and the video on the right shows the same flap eight hours later. Redness decreased and capillary refilling time increased, suggesting that flap hyperemia had resolved.
