# Supplementary material for: Predictors, management and prognosis of initial hyperemia of free flap
Source: Sci Rep. 2024 Feb 16;14:3894. doi: 10.1038/s41598-024-53834-2 (PMC10873382; doi:10.1038/s41598-024-53834-2)
Supplement: Supplementary file 2 — Supplementary Information 2. [file 41598_2024_53834_MOESM2_ESM.docx]

**Supplementary table 1. Intraoperative fluid therapy of the two groups**

|  | Overall  (N = 204, 100%) | Hyperemia  (N = 35, 17.2%) | No Hyperemia  (N = 169, 81.8%) | p-value |
| --- | --- | --- | --- | --- |
| Transfusion | 23 (11.3%) | 4 (11.4%) | 19 (11.2%) | 1.000 |
| Red blood cells | 22 (10.8%) | 4 (11.4%) | 18 (10.7%) | 1.000 |
| Fresh Frozen Plasma | 2 (1.0%) | 0 (0.0%) | 2 (1.2%) | 1.000 |
| Cryoprecipitate | 1 (0.5%) | 0 (0.0%) | 1 (0.6%) | 1.000 |
| Plasma volume resuscitation (ml/h/kg) | 1.6 (1.2, 2.1) | 1.5 (1.2, 1.9) | 1.6 (1.2, 2.1) | 0.494 |
| Urine output (ml/h/kg) | 1.4 (1.0, 2.0) | 1.4 (0.8, 1.9) | 1.5 (1.0, 2.0) | 0.333 |
| Total fluid input – urine output (ml/h/kg) | 0.2 (-0.5, 0.7) | 0.1 (-0.2, 0.5) | 0.3 (-0.6, 0.7) | 0.706 |

**Supplementary table 2. Hemodynamic variables of the two groups**

|  | Hyperemia  (N = 35, 17.2%) | No Hyperemia  (N = 169, 81.8%) | p-value |
| --- | --- | --- | --- |
| Preoperative SBP | 123.5 (112.0, 131.0) | 126.5 (115.0, 138.3) | 0.112 |
| Preoperative MBP | 89.7 (79.0, 97.0) | 90.7 (84.3, 99.6) | 0.176 |
| Preoperative DBP | 73.5 (68.0, 80.0) | 74.0 (67.0, 81.3) | 0.658 |
| NIBP systolic | 103.0 (97.3, 115.1) | 104.8 (99.3, 113.7) | 0.846 |
| NIBP mean | 79.1 (75.1, 84.4) | 79.2 (73.4, 85.9) | 0.911 |
| NIBP diastolic | 67.8 (64.0, 72.0) | 67.0 (60.2, 73.0) | 0.739 |
| ABP systolic | 110.9 (100.5, 114.2) | 109.3 (103.5, 116.4) | 0.764 |
| ABP mean | 80.5 (73.9, 86.1) | 76.5 (73.6, 82.4) | 0.089 |
| ABP diastolic | 62.0 (58.3, 70.0) | 58.9 (55.6, 63.8) | 0.020 |
| *Delta NIBP systolic | -19.5 (-29.1, -6.0) | -19.5 (-32.2, -6.7) | 0.374 |
| *Delta NIBP mean | 76.8 (-6.6, 82.8) | 74.0 (0.0, 83.0) | 0.987 |
| *Delta NIBP diastolic | -5.0 (-10.5, 0.4) | -7.5 (-14.9, 3.2) | 0.377 |
| *Delta ABP systolic | -13.5 (-24.0, -5.7) | -16.9 (-29.0, -6.0) | 0.340 |
| *Delta ABP mean | 76.7 (0.0, 83.3) | 74.6 (5.9, 80.5) | 0.365 |
| *Delta ABP diastolic | -12.5 (-20.0, -5.6) | -13.5 (-20.3, -7.4) | 0.312 |
| Temperature | 35.8 (35.5, 36.1) | 35.7 (35.4, 36.0) | 0.498 |

SBP: Systolic blood pressure, MBP: Mean blood pressure, DBP: Diastolic blood pressure, NIBP: Non-invasive blood pressure, ABP: Arterial blood pressure, *: Blood pressure difference from preoperative levels
